# Supplementary material for: The Role of Ultrasound in the Preparation of Zein Nanoparticles/Flaxseed Gum Complexes for the Stabilization of Pickering Emulsion
Source: Foods. 2021 Aug 25;10(9):1990. doi: 10.3390/foods10091990 (PMC8468403; doi:10.3390/foods10091990)
Supplement: Supplementary file 1 [file foods-10-01990-s001.zip › foods-1324036-supplementary.pdf]

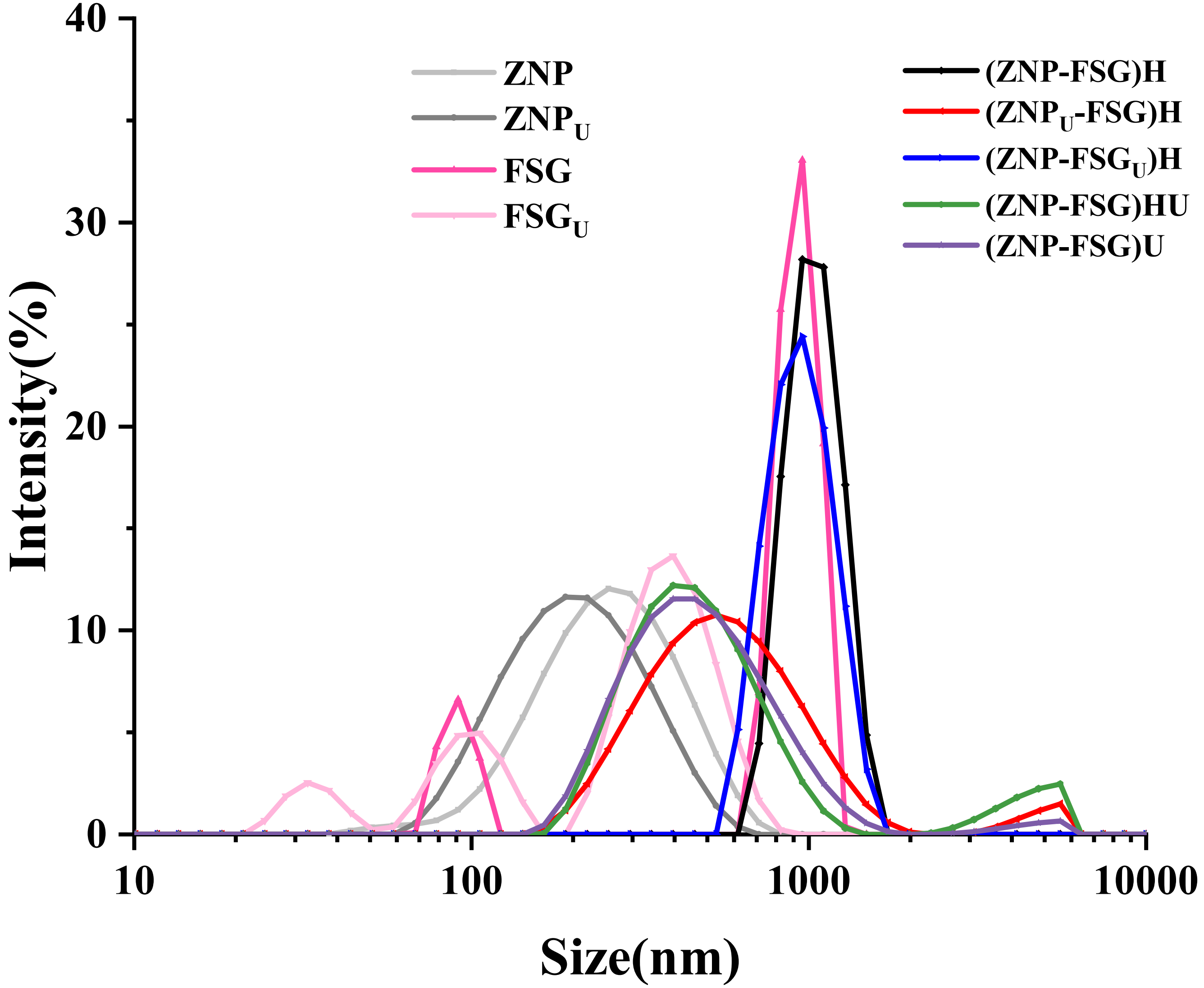

Figure S1 The particle size distribution of 9 various ingredients or ZNP -FSG complexes subjected to various ultrasound and homogenization treatments
